# The Role of the Endophytic Microbiome in the Grapevine Response to Environmental Triggers

**DOI:** 10.3389/fpls.2019.01256

**Published:** 2019-10-09

**Authors:** Davide Pacifico, Andrea Squartini, Dalila Crucitti, Elisabetta Barizza, Fiorella Lo Schiavo, Rosella Muresu, Francesco Carimi, Michela Zottini

**Affiliations:** ^1^Institute of Biosciences and BioResources (IBBR), National Research Council of Italy (CNR), Corso Calatafimi, Palermo, Italy; ^2^Department of Agronomy, Food, Natural Resources, Animals and the Environment, University of Padua, Legnaro, Italy; ^3^Department of Biology, University of Padova, Padova, Italy; ^4^Institute for the Animal Production System in Mediterranean Environment (ISPAAM), National Research Council (CNR), Sassari, Italy

**Keywords:** *Vitis vinifera*, endophytes, plant growth-promoting bacteria, stress-tolerance, biocontrol

## Abstract

Endophytism within *Vitis* represents a topic of critical relevance due to the multiple standpoints from which it can be approached and considered. From the biological and botanical perspectives, the interaction between microorganisms and perennial woody plants falls within the category of stable relationships from which the plants can benefit in multiple ways. The life cycle of the host ensures persistence in all seasons, repeated chances of contact, and consequent microbiota accumulation over time, leading to potentially high diversity compared with that of herbaceous short-lived plants. Furthermore, grapevines are agriculturally exploited, highly selected germplasms where a profound man-driven footprint has indirectly and unconsciously shaped the inner microbiota through centuries of cultivation and breeding. Moreover, since endophyte metabolism can contribute to that of the plant host and its fruits’ biochemical composition, the nature of grapevine endophytic taxa identities, ecological attitudes, potential toxicity, and clinical relevance are aspects worthy of a thorough investigation. Can endophytic taxa efficiently defend grapevines by acting against pests or confer enough fitness to the plants to endure attacks? What are the underlying mechanisms that translate into this or other advantages in the hosting plant? Can endophytes partially redirect plant metabolism, and to what extent do they act by releasing active products? Is the inner microbial colonization necessary priming for a cascade of actions? Are there defined environmental conditions that can trigger the unleashing of key microbial phenotypes? What is the environmental role in providing the ground biodiversity by which the plant can recruit microsymbionts? How much and by what practices and strategies can these symbioses be managed, applied, and directed to achieve the goal of a better sustainable viticulture? By thoroughly reviewing the available literature in the field and critically examining the data and perspectives, the above issues are discussed.

## Introduction

Plant microbial endophytism represents a wealth of interactive relationships that are widespread in nature and often rely on mutual benefits due to plant growth-promoting (PGP) phenotypes. In some cases, the beneficial effect is directly related to microbial metabolism, such as nitrogen fixation, phytohormone production, phosphate solubilization, and pathogen suppression; or conversely, such benefits can be mediated by the stimulation of specific activities of the host plant, leading to increased enzymatic catalysis, and enhanced water and nutrient uptake or defense responses. Moreover, endophytic microorganisms occupy an ecological niche that overlaps that of many phytopathogens, and their exploitation as biocontrol agents (BCAs) represents a possible strategy to reduce the use of pesticides in vineyards (Compant et al., 2013). Much of the applicative potential of agriculture and biotechnology is derived from plant–microbe interactions, but some requisites are the isolation of *ex planta* and multiplication of useful strains. According to some authors, this condition appears proportionally frequent compared with the situation observed in bulk soil or water environments (Finkel et al., 2017). However, other authors report the opposite and note the considerable proportion of non-plate-culturable endophytes (Thomas et al., 2017). West et al. (2010) recorded a certain degree of difference between culturable endophytic communities and those obtained by culture-independent approaches, suggesting a non-negligible level of non-culturability among endophytes. This limitation has been confirmed by further studies, which testified a major issue of recalcitrance to cultivation in grapevine endophytic bacteria (Thomas et al., 2017). When amenable to growth, selected members of the inner microbiota can be exploited as inoculants to foster plant growth in agriculture, horticulture, and silviculture or used for soil or water decontamination, such as in phytoremediation applications (Santoyo et al., 2016).

**Figure 1:**
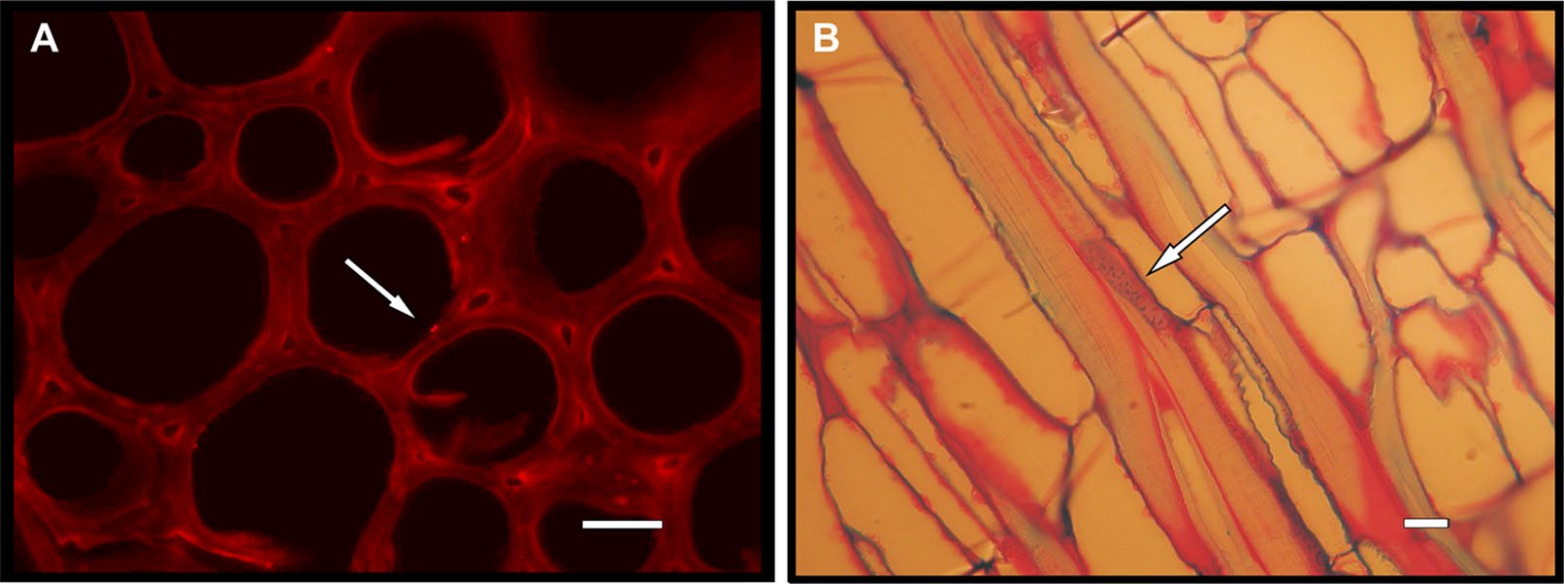
Endophytes inside plant tissues detected trough fluorescence microscopy techniques. **(A)** Epifluorescence image on transverse sections of a *Centaurea horrida* stem stained by propidium iodide. Arrow: endophytic bacterium cell adhering to a vessel within the plant’s vascular system. Bar = 15 μm. **(B)** Optical microscopy of basic fuchsin/astra blue double-stained longitudinal thin sections from a *Hedysarum glomeratum* root in proximity to the crown. Endophytic bacteria (indicated by the arrow) are distinguishable within a xylem vessel. Bar = 10 μm.

The precise identification of endophytic microorganisms is necessary to determine their distribution in host plants and their beneficial effects. The traditional methods used for the identification of BCAs are based on isolation and culturing on artificial media, followed by biochemical, immunological, and molecular characterization; however, the techniques of fluorescence microscopy can be used to detect endophytes inside plant tissues (**Figures 1A, B**). Over the last few years, advanced molecular techniques have allowed researchers to investigate the biodiversity of grapevine endophytes, particularly endophytic species that are slow growing or cannot be grown in axenic culture. Culture-independent methods, including automated ribosomal intergenic spacer analysis (ARISA) (Pancher et al., 2012), denaturing gradient gel electrophoresis (DGGE) (West et al., 2010), and single-strand conformation polymorphism (SSCP) analysis (Rezgui et al., 2016), can provide a general overview of the grapevine endophytic microbiome composition, although metagenomic approaches based on massive DNA sequencing of prokaryotic 16SrDNA or eukaryotic ITS1-5.8S-ITS2 rDNA genes are the methods of choice to assess the composition of grapevine endophytic communities (Pinto et al., 2014; Morgan et al., 2017; Dissanayake et al., 2018).

## Biodiversity

### Plant Influence on Endophytic Composition

Regarding the endophytic presence, a vast diversity of taxa has been documented in both cultivated (*Vitis vinifera* subsp. *vinifera*) and wild (*V. vinifera* subsp. *sylvestris*) grapevines. Different factors could shape the grapevine microbiome, as follows: seasonality, plant genotype, age, pedo-climatic features, surrounding wild plants, presence of pathogens, etc.

A survey of grapevine cultivar Glera plants from six different vineyards yielded 381 culturable bacterial isolates from the inner portions of surface-sterilized stems and leaves (Baldan et al., 2014). A large share of these bacteria (30%) belonged to the genus *Bacillus*, while the remainder included *Paenibacillus*, *Microbacterium*, *Staphylococcus*, *Micrococcus*, *Stenotrophomonas*, *Variovorax*, *Curtobacterium*, and *Agrococcus*. A certain degree of local specificity was reported as populations from different vineyards were rather different; in addition, seasonality was recorded, and differences were observed between the May and October samplings (Baldan et al., 2014).

In cultivated grapevine, the composition of bacterial communities is significantly associated with the rootstock genotype and shows different interactions; the nature of the rootstock was addressed as a possible variable explaining endophyte diversity. Marasco et al. (2018) investigated how different rootstocks could affect the recruitment of bacteria from the surrounding soil and found that bacterial community diversity and networking in the roots were profoundly influenced by the rootstock type. Interestingly, it has been observed that despite selecting different bacterial components, grapevine rootstock genotypes possess PGP traits similar to those exhibited by different bacteria that provide fundamental ecological services (Marasco et al., 2018).

The age of grapevine plants has been explored as a possible factor affecting endophytic community composition. Three-year-old and 15-year-old plants of the cv. Corvina were compared and found to be differentially invaded. Actinobacteria and Bacilli prevailed in the 3-year-old plants, while the older plants featured more Proteobacteria. Younger *Vitis* also have a higher diversity of taxa and a particular abundance of the *Rhizobium* genus, while the old plants contained higher shares of *Pantoea*. The phenotypes represented more in the 15-year-old plants were phosphate solubilization and 1-aminocyclopropane-1-carboxylate (ACC) deaminase activity (Andreolli et al., 2016).

Campisano et al. (2015) explored the difference between wild and cultivated grapevines in terms of endophytic communities; overall, 155 bacterial strains were isolated from 88 plants of the two types grown in the same climate. The species diversity resulted considerably higher in wild grapes than domesticated varieties with 25 genera versus six, respectively. In addition, regarding the multivariate ordination by phenotypes, the clustering was distinct to the isolates from the wild or cropped specimens, even when the bacteria belonged to the same genus.

An important question in this type of study is as follows: to what extent is a plant-associated microbiome specific to its host compared with the surrounding vegetation? The degree of overlap in bacterial communities isolated from grapevines and those found in weeds sharing the same vineyard has been analyzed (Samad et al., 2017). Assuming that the same soil offers the different coexisting plants a pool of microbes from which to choose, the authors identified the rhizosphere and inner root bacterial checklists of *V. vinifera* and those of four herbaceous plants, including three annual therophytes (*Stellaria media*, *Veronica arvensis*, and *Lamium amplexicaule*) and one perennial (*Lepidium draba*). The analysis was carried out using culture-independent 16S amplification and metabarcoding. The shared portion of the microbiome featured 145 operational taxonomic units (OTUs) in the rhizosphere, and only nine belonged to the truly endophytic contingent isolated from inside the roots. The bacteria associated with the weeds had a higher diversity in the rhizosphere than those related to the grapevine, but the endophytic subgroup displayed more diversity in the perennial plants (*Vitis* or *Lepidium*). The PGP traits were more represented in the isolates from the weeds. Overall, it appears that even plants sharing the same soil are characterized by significantly different microbiomes both at the rhizosphere level and inside the roots, but in the latter compartment, their differences are more pronounced.

**Figure 2:**
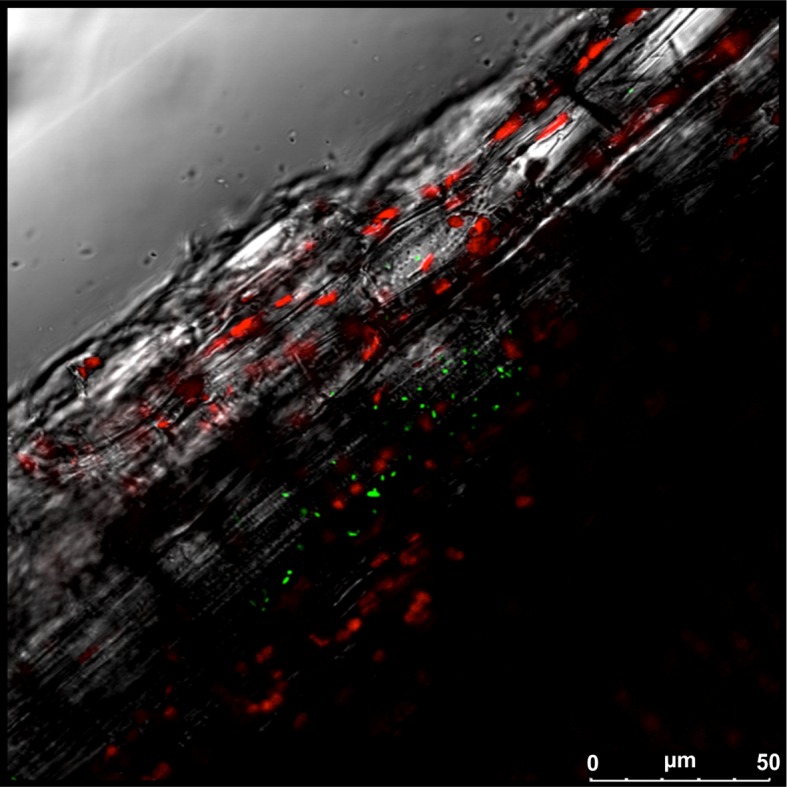
*Bacillus licheniformis* colonizing a grapevine plant. Laser scanning confocal microscopy of stem sections of Glera cuttings inoculated with *Bacillus licheniformis* GL174::gfp2x. Overlay of green fluorescent protein (GFP) signal (green), chlorophyll (red), and bright field.

The interaction between endophytes and plants occurs in different areas, including the root and foliar surfaces. Regarding the provenance of the endophytic microbiota and its way of entry into plants, it is generally assumed that the main route is from soil to roots with endophytes ascending to the epigeal parts or alternatively through gaps along the plant overall surface, including wounds or stomata (Compant et al., 2010). Once endophytes enter into the plant, they move systemically (Nigris et al., 2018), spreading in different tissues as shown in **Figure 2**, where green fluorescent protein (GFP)-tagged *Bacillus licheniformis* can be visualized within the stem of a grapevine-inoculated plant. Indeed, endophytes can colonize other tissues and organs, including reproductive organs, thus allowing their transfer through the vascular system (Lidor et al., 2018) or apoplastic spaces (Compant et al., 2011). An analysis of different aerial portions of grapevines was carried out and revealed differential colonization by endophytes; both *Pseudomonas* and *Bacillus* were found within the flower and ovules, while only the latter was present in the berries and seeds (Compant et al., 2011). Using DGGE and fatty acid methyl ester profiling, some authors have also documented the possibility that grapevine epiphytes originally dwelling on the external surface of leaves and stems could become endophytes when windows of opportunity for such internalization arise (West et al., 2010). However, there are documented exceptions that add complexity to the picture. In grapevines, the sap-feeding leafhopper pest *Scaphoideus titanus*, which is recognized as the vector for phytoplasma diseases, has also been found to be capable of transferring various species of endophytic bacteria from one grapevine plant (source plant) to another (sink plant) (Lòpez-Fernàndez et al., 2017); the acquired taxa included members of the phyla Proteobacteria, Actinobacteria, and Firmicutes. While the insect spends time exclusively on leaves or stems, in sink plants, the root endophytic communities acquired a composition very close to that found within the insect vector. These data suggest that the insect-mediated exchange of endophytes among plants could be an important and still overlooked component of the mechanisms underlying these plant–microbe interactions. However, an even more extreme example of the cross-transfer of microbes to plants concerns the taxon *Propionibacterium acnes*, which has been shown to have been acquired by grapevine in an inter-kingdom transmission from humans (Campisano et al., 2014a). Such occurrence is reminiscent of several cases in which bacterial host switches were traced from domestic animals to humans, but here, the case is particularly remarkable as it could involve the opposite direction and a member of the plant kingdom as a recipient sink.

A further factor conducive to differences in the endophytic communities of grapevine is the presence of pathogens, such as *Agrobacterium vitis*, which is the agent of grapevine crown gall disease (Faist et al., 2016). The authors have found the differences in the microbial community occurring at the graft union as discriminative. In particular, the microbial community from plants without crown gall disease showed a transient composition, changing from across seasons, while that from plants with crown galls had a higher species diversity and regularly featured the dominance of the same three taxa, including *Pseudomonas* sp. *A. vitis* and a member of the Enterobacteriaceae family (Faist et al., 2016). The diversity and abundance of endophytes have been investigated and compared between healthy grapevine and plants affected by flavescence dorée (FD) and bois noir phytoplasmas while considering the seasonal variation (Bulgari et al., 2011). The authors sampled during three different time points and reported that independent of the sampling period, phytoplasmas can shape endophytic bacterial communities by enriching such communities with strains able to elicit plant defense responses. Genera associated with the infection process include some that have been reported to be associated with biocontrol action, such as *Burkholderia*, *Methylobacterium*, *Sphingomonas*, and *Pantoea*. Overall, the communities found in the healthy grapevine featured a higher diversity than those in the diseased plants. Endophytes and plant pathogens can indeed coexist within the same host and result in evolutionary paths that diverge at a certain stage. A study concerning grapevine invaded or not invaded by the black fungi *Aureobasidium pullulans* and *Epicoccum nigrum* reported that their presence did not result in detectable changes in the associated bacterial populations (Grube et al., 2011).

A different approach to studying endophytism relies on genomic data to search for genes that could distinguish endophytic strains from pathogenic strains belonging to closely related lineages. This type of survey was carried out in the genomes of seven endophytes isolated from grapevine, which were compared with members of the same genera but involved in pathogenic interactions (Lòpez-Fernàndez et al., 2015). Their comparison showed that endophytes and pathogens have a high number of common virulence-related genes in their core pangenome with a high degree of conservation in each genus regardless of the endophytic or pathogenic lifestyles. Therefore, the structural organization of endophyte genomes is reflective of the conservation of properties spanning over different attitudes, including pathogenicity. Through a bioinformatics approach, it is possible to identify particular genes in endophyte genomes strictly associated with the endophytic lifestyle, which can contribute to the peculiar recognition of beneficial microorganisms (Ali et al., 2014).

### Environmental Influence on Endophytic Diversity

The microbial component in viticulture and oenology has been increasingly recognized to have a major imprint on the regional terroir. A complex of environmental variables is linked to the geographical origin and its ensuing endophytic community composition. Studies have shown that the microbiome involved during the early fermentation stages, which is partially determined by endophytic plant-borne yeasts and bacteria, complies with a well-delineated biogeography reflecting the signatures of different winegrowing regions with an additional but minor influence from the grape variety and vintage year (Bokulich et al., 2014; Gilbert et al., 2014). Notwithstanding, the soil is a main reservoir for endophyte recruitment, and a strong selection process appears to be operative in favor of strains endowed with relevant PGP phenotypes, as the final community is not merely a mirror of the soil array of microbiota but rather a distinct sub-community (Novello et al., 2017). As the terroir concept implies, soil is ultimately regarded as a major environmental factor conditioning the microbial populations that are eventually associated with their host plants. This issue has been thoroughly addressed, and the factors particularly critical for the determination of grapevine inner microbiota include the soil pH, soil carbon, and C:N ratio; most taxa associated with grapevine organs originated in soil and had a marked degree of local vineyard scale specificity (Zarraonaindia et al., 2015). Among the environmental conditions, soil pH is generally recognized as a major driver shaping bacterial communities. Its effect in modelling those interacting with grapevine was addressed in plants grown in acidic or alkaline soils (Karagöz et al., 2012); in total, 27 genera were identified; overall, gram-negative taxa were dominant in both soil types, and *Pseudomonas* and *Bacillus* were recurring genera. The observed differences included that *Acidovorax delafieldii*, *Pseudomonas mendocina*, *Aeromonas ichthiosmia*, *Hafnia alvei*, *Raoultella terrigena*, *Paenibacillus alginolyticus*, *Arthrobacter aurescens*, *Kocuria kristinae*, *Curtobacterium flaccumfaciens*, *Flavobacterium johnsoniae*, and *Sphingobacterium spiritivorum* were isolated only from alkaline sites. In contrast, *Ralstonia eutropha*, *Citrobacter amalonaticus*, *Enterobacter hormaechei*, *Arthrobacter globiformis*, *Rhizobium rubi*, *Paracoccus denitrificans*, *Pseudomonas syringae*, *Citrobacter freundii*, *Serratia odorifera*, *Arthrobacter oxydans*, *Kocuria rosea*, and *Brevibacterium epidermidis* were isolated only from the acidic soils.

Other studies addressed pest management (organic vs. integrated) as a variable and determined its effect on the bacterial endophyte community of Merlot and Chardonnay grapevine cultivars (Campisano et al., 2014b). The results indicated that while *Ralstonia*, *Burkholderia*, and *Pseudomonas* were present in all samples, the relative abundance of the taxa defined the consistent differences, and the genera *Mesorhizobium*, *Caulobacter*, and *Staphylococcus* were found more frequently in the organically managed vineyards, while *Ralstonia*, *Burkholderia*, and *Stenotrophomonas* were abundant in the vineyards where integrated pest management was operative. In this respect, the differences related to the cultivar were minor compared with those ascribed to the management practices. Pancher et al. (2012) showed that the possibility of differentiating communities in relation to management or the cultivar also depends on the technique used. The fungal taxa detected in a parallel analysis of endophytic fungi in the same two grapevine cultivars included *Absidia glauca*, *Pennicillium restrictum*, *Alternaria* sp., *Botrytis cinerea*, *Fusarium* sp., *Trichoderma reesei*, *Fusarium oxysporum*, *Neurospora crassa*, *Pennicillium chrysogenum*, *Podospora anserina*, and *Davidiella tassiana*; the culture-dependent methods yielded overlapping results, while the molecular DNA-based approaches had sufficient power to resolve the differences between the organic and integrated pest managements.

## Beneficial Effects of Grapevine Endophytes on Abiotic Stress Tolerance

**Figure 3:**
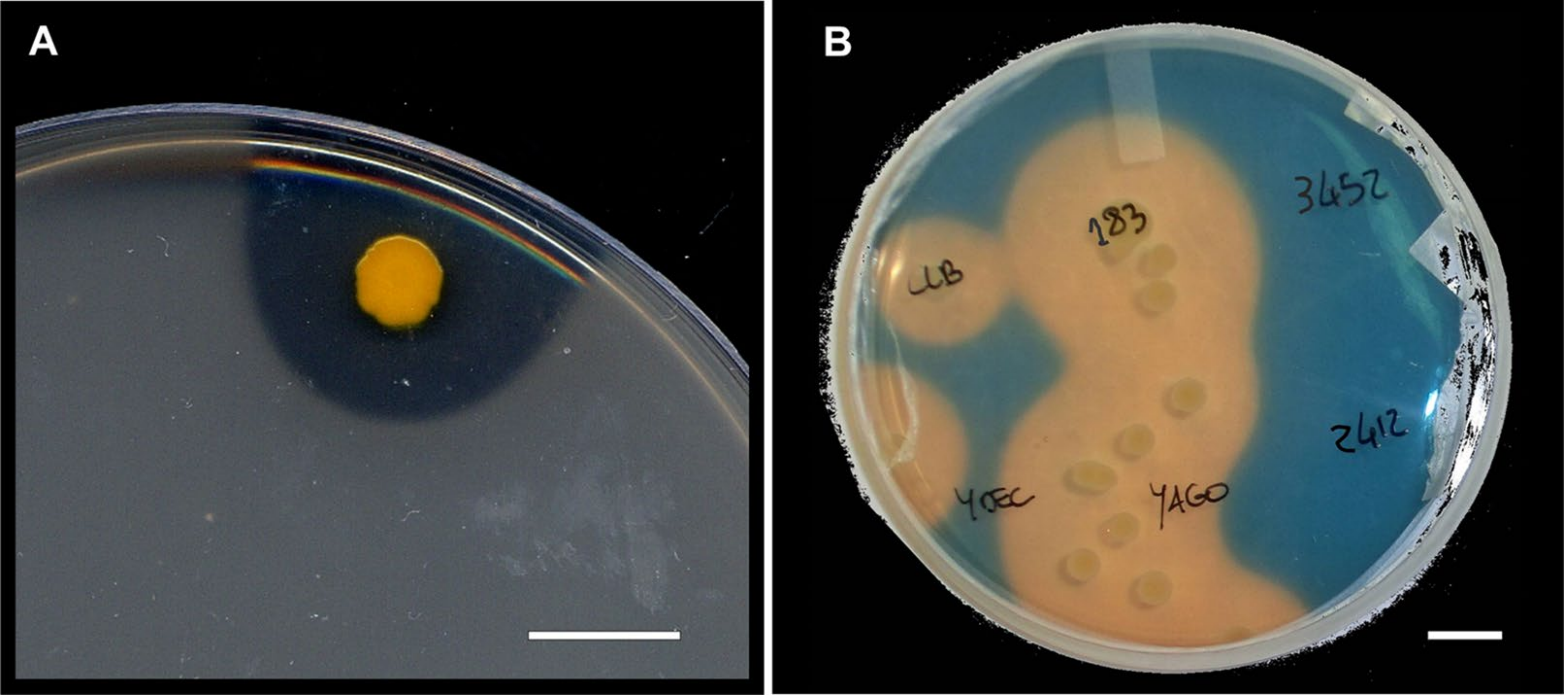
Isolated grapevine endophytes. **(A)** Grapevine endophyte showing antibiosis production towards a layer of *Bacillus subtilis* on a petri dish. Bar = 1 cm. **(B)** Grapevine endophytes, including some showing positivity (discoloring to yellow background in the Chrome Azurol-S assay) to an iron-solubilization test for siderophores. Bar = 1 cm.

Endophytes employ different mechanisms to exert beneficial effects on plant growth or biotic/abiotic stress resistance, and these mechanisms act either directly by releasing plant hormones, secondary metabolites (**Table 1**), biocides, or antibiotics (an example is shown in **Figure 3**) or indirectly by modifying the plant physiology and nutrient balance, leading to a reduced susceptibility to diseases or abiotic stresses (Lugtenberg and Kamilova, 2009; Compant et al., 2013).

**Table 1:** Potential roles and mechanisms of main endophytes involved in biotic and abiotic stress tolerance.

BIOTIC STRESS			
Pathogen	Endophytes	Mechanism associated with the tolerance	Reference
*Botrytis cinerea*	*Acinetobacter lwoffii* (PTA-113 and PTA-152), *Pseudomonas fluorescens* (PTA-268 and PTA-CT2), *Pantoea agglomerans* (PTA-AF1 and PTA-AF2), *Bacillus subtilis* (PTA-271)	Induced the activities of lipoxygenase (LOX), phenylalanine ammonia-lyase (PAL), β-1,3 glucanase, and chitinase. Induced the oxidative burst. Accumulated the stress-related metabolites phytoalexin (trans resveratrol and trans-ε-viniferin).	Magnin-Robert et al. (2007)*Eur. J. Plant Pathol*. 118, 43–57. Trotel-Aziz et al. (2008)*Environ. Exp. Bot*. 64(1), 21–32 Verhagen et al. (2011)*Phytophatol*. 101, 768–777.
	*P. fluorescens* PTA-CT2	Regulated the expression of defense-related genes in leaf and root, including those with transcriptional factor functions (*JAZ9*, *NAC1*, and *ERF1*), of secondary metabolism (*PAL*, *STS*, *LOX9*, *ACCsyn*, *GST*, *CHS*, *CHI*, *LAND*, and *ANR*), and PR proteins (*PR1*, *PR2*, *PR3*, *PR5*, and *PR6*).	Gruau, C. et al. (2015) *Mol. Plant Microbe Interact*. 28, 1117–1129.
	*Ulocladium atrum*	Enhanced chitinase activity.	Ronseaux et al. (2013) *Agronomy* 3, 632–647.
	*Burkholderia phytofirmans* PsJN	Induced callose deposition and H_2_O_2_ production. Primed the expression of *PR1*, *PR2*, *PR5*, and *JAZ* in bacterized plantlets after pathogen challenge. Modulated the carbohydrates metabolism.	Miotto-Vilanova et al. (2016)*Front. Plant Sci*. 7:1236.
	*B. subtilis* (BBG127, BBG131 Bs2504, and BBG125)	Treatment with *B. subtilis* strains non-producing lipopeptides, overproducing surfactin, overproducing plipastatin, and overproducing mycosubtilin differentially activated the plant innate immune response. Modulated genes encoding a chitinase (*chit4c*), a protease inhibitor (*pin*), a salicylic acid (SA)-regulated marker (*Vv17.3*), and a glucanase (*gluc*).	Farace et al. (2015)*Mol. Plant Pathol*. 16, 177–187.
	*Microbacterium imperiale Rz*19M10, *Kocuria erythromyxa* Rt5M10, *Terribacillus saccharophilus* Rt17M10	Induced a systemic response that triggers increases on monoterpenes, sesquiterpenes, tocopherols, and membrane sterols (enhanced antioxidant capacity).	Salomon et al. (2016)*Plant Physiol. Biochem*. 106, 295–304.
	*Streptomyces anulatus* S37	Induced rapid and transient generation of H_2_O_2_, extracellular alkalinization, and an activation of two mitogen-activated protein kinase (MAPKs) followed by the expression of *LOX9*, *PAL*, *STS*, and *GST* genes in primed cells. Induced defenses modulated by Ca^2+^ signaling.	Vatsa-Portugal et al. (2017)*Front. Plant Sci*. 8:1043.
*B. cinerea, Plasmopara viticola, Xiphinema index* Nematodes	*Paenibacillus sp*. strain B2	Modulated the expression of defense-related genes *CHI, PAL, STS, GST*, and *LOX* and pathogenesis-related protein PR-6. Reduced nematode root infection associated with the expression of genes resistant to nematodes *Hero* and *Hs1^pro−1^*.	Hao et al. (2017)*Biological Control* 109, 42–50.
*Rhizobium vitis* (Ti) VAT03-9 (tumorigenic)	*R. vitis* ARK-1	Co-inoculation of ARK-1 with a Ti strain VAT03-9 into grapevine shoots suppressed the expression of the virulence genes virA, virD3, and virG of VAT03-9.	Kawaguchi et al. (2019)*BMC Res. Notes*. 12:1.
Flavescence dorée phytoplasma	*Pseudomonas migulae* 8R6	Production of 1-aminocyclopropane-1-carboxylate (ACC) deaminase enzyme helps the plant to regulate the level of the stress-related hormone ethylene	Gamalero et al. (2017)*Plant Biosyst*. 151, 331–340.
*Diplodia seriata* (strains F98.1 and Ds99.7) (botryosphaeria dieback)	*Aureobasidium pullulans* strain Fito_F278	Modulated genes encoded for plant defense proteins: PR protein 6 (*PR6*) and *β-1,3-glucanase* (*Gluc*); detoxification and stress tolerance: haloacid dehalogenase hydrolase (*Hahl*), α-crystalline heat shock protein (*HSP*), *β-1,3-glucanase* (*GST5*); phenylpropanoid pathway: (*STS*) cell wall (*fascAGP*) and water stress (*Pip 2.2*).	Pinto et al. (2018)*Front. Microbiol*. 9:3047.
*Neofusicoccum parvum* (botryosphaeria dieback)	*B. subtilis* PTA-271	Antagonized N. parvum by delaying its mycelial growth and detoxifying both (R)-mellein and (−)-terremutin. Primed defense genes including PR2 (a β-1,3-glucanase), NCED2 (involved in ABA synthesis), and PAL at systemic level after pathogen inoculation.	Trotel-Aziz et al. (2019)*Front. Plant Sci*. 10:25.
*Phaeomoniella chlamydospora* (trunk diseases)	*Paenibacillus sp*. S19 *Bacillus pumilus* S32	Induced resistance against trunk disease fungi: induced the expression of defense-related genes: *PR1, PR10, CHIT3, PAL, STS, CHS, ANTS, CALS, GST*, and *GLU*.	Haidar et al. (2016b)*Microbiol. Res*. 192, 172–184.
ABIOTIC STRESS			
STRESS	Endophytes	Mechanism associated with the tolerance	Reference
Chilling	*B. phytofirmans strain* PsJN	Elevated the stress-related metabolites (proline, aldehydes, malondialdehyde, and phenolics). Enhanced the rate of photosynthesis and starch deposition. Induced the expression of the defense genes: StSy, PAL, Chit4c, Chit1b, Gluc, and LOX. Induced the expression of trehalose-related genes *TPS1*, *TPPA*, and *TRE*. Accumulated T6P and trehalose.	Barka et al. (2006)*Appl. Environ. Microbiol*. 72, 7246–7252. Theocharis et al. (2012)*Mol. Plant Microbe Interact*. 25, 241–249. Fernandez et al. (2012)*Planta* 236, 355–369.
Drought stress	*Bacillus licheniformis* Rt4M10 *P. fluorescens* Rt6M10	Induced synthesis of monoterpenes and sesquiterpenes. Produced and induced synthesis of ABA.	Salomon et al. (2014)*Physiol. Plant*. 151, 359–374.
Salt or drought stress	*Bacillus amyloliquefaciens* SB-9	Secreted and produced melatonin and three intermediates of the melatonin biosynthesis pathway: 5-hydroxytryptophan, serotonin, and *N*-acetylserotonin. Increased the upregulation of melatonin synthesis, as well as that of its intermediates, but reduced the upregulation of grapevine tryptophan decarboxylase genes (TDCs) and a serotonin *N*-acetyltransferase gene (SNAT). Reduced the production of malondialdehyde and reactive oxygen species (H_2_O_2_ and O_2_^−^) in roots.	Jiao et al. (2016)*Front. Plant Sci*. 7:1387.
	*Pseudomonas fluorescence* RG11	Enhanced endogenous melatonin in plants. Regulated melatonin-related genes: *TDC1* (putative tryptophan decarboxylase-1) and *SNAT* (serotonin *N*-acetyltransferase).	Ma et al. (2017)*Front. Plant Sci*. 7:2068.
Arsenic contamination	*Micrococcus luteus, B. licheniformis, P. fluorescens*	Increased antioxidant enzyme activity (APX, ascorbate peroxidase; CAT, catalase; and POX, peroxidases activity). Reduced peroxidation of membrane lipids (reduced malondialdehyde content) and photosystems damage membrane damage in As presence.	Funes Pinter et al. (2017)*Appl. Soil Ecol*. 109, 60–68. ) *Agr. Ecosyst. Env*. 267, 100–108.
High temperature and drought stress	Bioradis Gel (Bioera SLU, Tarragona, Spain): mixture of five AMF fungi (*Septglomus deserticola, Funneliformis mosseae, Rhizoglomus intraradices, Rhizoglomus clarum, and Glomus aggregatum*), and a mixture of rhizobacteria belonging to the *Bacillus* and *Paenibacillus* genera	Under elevated temperature and deficit irrigation, inoculated plants reached higher berry anthocyanins and evidenced some modifications in berry ABA catabolism.	Torres et al. (2018)*Plant Sci*. 274, 383–393.

Abiotic stresses, such as heat, salinity, and drought, trigger trehalose synthesis in plants, which can provide the plants stress endurance. Trehalose forms a gel during cellular dehydration, preventing excessive water loss due to its high stability against acids and heat. Inoculation with the *Paraburkholderia phytofirmans* strain PsJN has been observed to be conducive as a stress-preventive trait as plants bearing this endophyte began to accumulate trehalose and its intermediate trehalose-6-phosphate (T6P) at 26°C (Fernandez et al., 2012). In particular, it has been reported that *P. phytofirmans* can affect trehalose metabolism in grapevine plants through the stimulation of T6P synthesis or the inhibition of T6P degradation (Esmaeel et al., 2018). A deep analysis of 48 genomes of *Burkholderia* species revealed that 161 clusters involved in secondary metabolite synthesis were likely involved in signaling between grapevine and endophytic bacteria (Esmaeel et al., 2016). It has been demonstrated that *P. phytofirmans* sets grapevine metabolism to a state of alert, enabling rapid and intense resistance responses to subsequent stresses. In this case, the benefit provided by endophytes does not occur directly through the induction of stress-related genes but rather occurs by preparing the plant to activate a faster and stronger defense response upon stress (Esmaeel et al., 2016). In addition, *P. phytofirmans* can induce different *PR* genes [encoding chitinase, phenylalanine ammonia-lyase (PAL), lipoxygenase (LOX), and glucanase], protecting the plant from low temperatures (Theocharis et al., 2012). In addition, the endophyte is able to induce genes coding enzymes involved in reactive oxygen species (ROS) scavenging; a decrease in the ROS concentration is important because it converts detoxification compounds into signaling molecules.

A different major factor of stress in most plants is drought. Soil microorganisms have different strategies to cope with limited water potential, including the accumulation of compatible solutes (glycine-betaine, proline, trehalose, and exopolysaccharide production) and exopolysaccharide production (Ngumbi and Kloepper, 2016). Their phenotypes have been shown to be transferrable to the plants that they can invade as endophytes (Mayak et al., 2004; Timmusk et al., 2014). Regarding grapevine, an extensive analysis of endophytes from different rootstocks and the cv. Barbera in relation to drought has been performed (Rolli et al., 2015). In this study, eight strains from the *Pseudomonas*, *Acinetobacter*, *Bacillus*, *Delftia*, and *Sphingobacterium* genera were selected from over 510 total isolates and shown to be capable of colonizing different hosts, such as *Arabidopsis*. It has been demonstrated that different strains of the *Pseudomonas* and *Acinetobacter* genus and *Bacillus subtilis* could alleviate drought damage because of their root/shoot growth-promoting activities (Rolli et al., 2015; Rolli et al., 2017). These bacteria were characterized by the production of indole acetic acid (IAA), ACC deaminase activity, phosphate solubilization, and ammonia production *via* peptone mineralization. Using the same isolates, in pepper plants, the authors demonstrated that the PGP activity phenotypes related to drought were not constitutive but underwent activation *in planta* when the host was challenged with drought stress. Drought-sensitive rootstocks displayed the most enhanced effects. Three strains were further tested on grapevine under outdoor conditions and conferred higher root biomass to the plants (Rolli et al., 2015). The retardation of water loss in *Vitis vinifera* was also demonstrated by root isolates of *Bacillus licheniformis* and *Pseudomonas fluorescens*, whose effects, as measured from 30 days after the inoculation of *in vitro*-cultured plants, also extended to other measurable and possibly related parameters. These parameters included the accumulation of abscisic acid (ABA) (stimulated 76-fold over the untreated control by *B. licheniformis* and 45-fold by *P. fluorescens*) and synthesis of defense-related terpenes, such as α-pinene, terpinolene, 4-carene, limonene, eucalyptol, lilac aldehyde A, α-bergamotene, α-farnesene, nerolidol, and farnesol (Salomon et al., 2014). Regarding *sensu lato*, endophytic grapevine drought stress has also been shown to be highly alleviated by the presence of mycorrhizal fungi, especially in plants grafted on drought-sensitive rootstocks, such as 775P, 101-14Mgt, and 5BB (Nikolaou et al., 2003). A related stress factor in plants is soil salinity, and in this case, the recruitment of microorganisms under the endophytic condition is a critical aspect (Numan et al., 2018). The mechanisms by which internalized microbes could help alleviate salt stress appear to be linked to their production of plant hormones, including auxins, gibberellins, and ABA (Yanni et al., 2001; Jha and Subramanian, 2013).

Many endophytes have the ability to synthetize ACC deaminase, which alleviates plant stress consequences, as it contributes to lowering the ethylene level. Ethylene is an important plant hormone that is extensively studied as a mediator of plant stress response signaling. Ethylene is formed from methionine *via S*-adenosyl-l-methionine, which is converted into 1-aminocyclopropane-1-carboxylic acid by the enzyme ACC oxidase. The stress-induced accumulation of ethylene is usually deleterious to plant growth and health. Several endophytes, such as *P. fluorescens*, *P. phytofirmans*, and *Pseudomonas migulae*, possess the ability to produce the enzyme ACC deaminase, which, by cleaving the ethylene precursor, avoids ethylene accumulation, masking drought and salinity stress and, thus, improving stress tolerance and plant growth (Miliute et al., 2015; Gamalero et al., 2017).

Other molecules are released by endophytes that can intervene in ROS scavenging and, thus, help grapevine cope with different abiotic stresses, such as cold, drought, or salinity. *B. licheniformis* has been demonstrated to release several types of secondary metabolites, such as monoterpenes, exerting an antioxidant activity, and sesquiterpenes, showing antimicrobial properties (Salomon et al., 2014). The other secondary metabolites in the terpenoid biosynthesis pathway are produced by endophytic microorganisms that could be important for their beneficial activity in grapevine. Indeed, *B. licheniformis* produces carotenoids that could act as antioxidant species, which is particularly helpful under different stress conditions (Cohen et al., 2018). Notably, these compounds are precursors of ABA in plants, serving as the base of the drought resistance conferred by *B. licheniformis* (Salomon et al., 2016). It has also been reported that *B. licheniformis* and *P. fluorescens* are able to induce the expression of the genes coding components of ABA synthesis and signaling pathways in grapevine plants (Salomon et al., 2014). Both bacterial endophytes and endophytic fungi (*Septglomus deserticola*, *Funneliformis mosseae*, *Rhizoglomus intraradices*, *Rhizoglomus clarum*, and *Glomus aggregatum*) could modulate ABA metabolism in inoculated grapevine plants, giving an advantage over uninoculated plants under drought conditions (Torres et al., 2018). It has also been reported that in grapevine, *B. licheniformis* enhanced ascorbate peroxidase activity, while *Micrococcus luteus* and *P. fluorescens* augmented peroxidase activity, exerting strong antioxidant activity (Funes Pinter et al., 2017).

Actually, many endophytes produce protective molecules, such as melatonin, proline, and carotenoids, which play an active defense role against abiotic stress. The action is not only local; in fact, endophytes can also produce volatile organic compounds (VOCs), which, in addition to being involved in the first interaction/recognition by the plant (Liu and Brettell, 2019), can also be precursors of important signal molecules. Interesting examples include the release of carotenoids in the host plant as precursors of hormones (ABA) or detoxifying enzymes, such as ACC deaminase, that modulate the ethylene content and consequently the damage triggered upon stress. An interesting example is melatonin accumulation, which occurs in *Bacillus amyloliquefaciens*-inoculated grapevines, counteracting the negative effects of salt, drought, and cold stress (Jiao et al., 2016). The same bacterial strain reduced the upregulation of tryptophan decarboxylase and serotonin *N*-acetyltransferase transcription (Jiao et al., 2016). Indeed, melatonin is a strong antioxidant that increases CuZn superoxide dismutase (SOD), Fe-SOD, catalase, and thylakoid‐bound ascorbate peroxidase activities (Baier et al., 2019). It has been observed that an enhancement of endogenous melatonin synthesis is triggered by various abiotic stress factors, which can also be due to endophytic bacteria, such as *P. fluorescens*, which is able to induce the transformation of tryptophan into melatonin in the roots of different grapevine cultivars (Ma et al., 2017). To achieve its beneficial effect, melatonin can be used in exogenous applications. However, melatonin-producing endophytes might have long-term effects on the endogenous melatonin levels in plants once they enter plant tissues. The presence of viruses in grapevine has been noted as a positive factor enhancing tolerance to water stress, shifting the role of some viruses from deleterious to mutualistic because of the long co-existence between the virus and its host (Gambino et al., 2012).

## Defense Against Biotic Agents

### General Aspects

*Vitis vinifera* can be affected by a numerous pathogens often associated with a severe reduction in yield and product quality (Armijo et al., 2016). To guarantee high-quality production, pesticides and fungicides are currently applied in vineyards, although the continuous use of chemicals may cause the emergence of resistant microorganisms, environmental pollution, and heavy consequences on human health (Pérez-Ortega et al., 2012; Damalas and Koutroubas, 2016).

Colonization by endophytic microorganisms places grapevine in a state of alert (primed) that allows a rapid and intense resistance response to subsequent stresses at a low fitness cost instead of wastefully activating defenses (Martinez-Medina et al., 2016; Mauch-Mani et al., 2017). Experimental evidence showed that endophyte perception triggers a local immune response that is significantly weaker in intensity than that occurring during non-host interactions (Bordiec et al., 2011; Trdá et al., 2014). This type of resistance can provide protection to the entire plant against a broad spectrum of fungal, oomycete, bacterial, and viral pathogens and insects (De Vleesschauwer and Höfte, 2009; Van der Ent et al., 2009; Pineda et al., 2010).

Most endophytic bacteria are well known for their capability to produce secondary metabolites that have an inhibitory effect on a wide range of phytopathogens. Some compounds are important for protection because they can inhibit the growth of other bacteria and fungi (antimicrobial compounds) (Shameer and Prasad, 2018), but they also play a significant role in the mechanisms underlying signaling, defense, and gene regulation. These metabolites comprise phytoalexins (Gruau et al., 2015) and various biocide compounds (Verhagen et al., 2011; Esmaeel et al., 2018), such as HCN (Samad et al., 2017) and antibiotics (Nigris et al., 2018; Shameer and Prasad, 2018). A common trait in the group of *Pseudomonas* and *Bacillus* is HCN production (Shameer and Prasad, 2018). HCN can act as a biocide, suppressing the growth of different pathogens, although in grapevine, it has been mainly studied due to its property of breaking summer bud dormancy (Sudawan et al., 2016). Several VOCs produced by different endophyte strains exhibit antibacterial and/or antifungal activity because they can reduce fungal growth, impair fungal spores and hyphae, and/or promote plant growth (Haidar et al., 2016b). An important strategy that helps endophyte-colonized plants cope with pathogens is the ability of endophytes to modulate plant metabolism to restrict pathogen growth and invasion (Miotto-Vilanova et al., 2016). Considering that many factors can influence the effectiveness of BCAs, their efficacy must be confirmed through appropriate experimental procedures both *in vitro* and *in vivo* possibly using a multi-organ screening approach (Haidar et al., 2016a). The organ host and pathogen genotype/strain may considerably affect the antagonist efficiency. Haidar et al. (2016a) reported that the antagonist efficiency of *Pantoea agglomerans* (S2 and S3) and *Enterobacter* sp. (S24) on *Botrytis cinerea* in grapevine leaves was lower than that observed in unwounded berries. Moreover, some endophytic species active against one tested pathogen could stimulate other diseases as reported in *Bacillus* sp. S43, which improved the symptoms caused by *Neofusicoccum parvum* when applied to grapevine cuttings against *B. cinerea* (Haidar et al., 2016a).

### Protection Against Bacterial Pathogens

Grapevine diseases caused by bacteria are characterized by a biotrophic host–pathogen relationship in which the microorganism interacts with the living plant without killing it. Crown gall caused by tumorigenic *Rhizobium vitis* is the most important bacterial disease of grapevine worldwide (Armijo et al., 2016). The biological control of crown gall disease in grapevine was achieved by the application of antagonistic endophytic bacteria, including *Enterobacter agglomerans*, *Rahnella aquatilis*, and *Pseudomonas* sp. (Bell et al., 1995), and the non-pathogenic *R. vitis* strains F2/5 (Burr and Reid, 1994) and ARK-1 (Kawaguchi, 2013). Moreover, endophytic *Pseudomonas fluorescens* 1100-6, *Bacillus subtilis* EN63-1, and *Bacillus* sp. EN71-1 isolated from *Malus domestica* were reported as potential BCAs of crown gall (Eastwell et al., 2006). The inhibition of tumorigenic *Rhizobium* spp. occurs through different mechanisms, such as the production of antibacterial compounds (Chen et al., 2007), quorum sensing, caseinolytic protease activation (Kaewnum et al., 2013), and suppression of virulence gene expression (Kawaguchi, 2015; Kawaguchi et al., 2019).

*Xylella fastidiosa*, which is the agent of grapevine Pierce’s disease (PD), is a gram-negative, xylem-limited bacterium transmitted by leafhopper vectors (Kyrkou et al., 2018). Due to the impact of *X. fastidiosa* infections, several attempts to identify potential BCAs against this pathogen have been published. Virulent strains of *X. fastidiosa* acting as an antagonistic of the wild-type strain reduced the severity of PD symptoms in the grapevine cv. Carignan (Hopkins, 2005). In particular, the *X. fastidiosa* strain EB92-1 isolated from elderberry provided good control of PD in vineyard of cv. Flame Seedless and cv. Cabernet Sauvignon (Hopkins, 2005). Newman et al. (2008) demonstrated that the incidence and severity of PD were significantly reduced in grapevine co-inoculated with *X. fastidiosa* and *Pseudomonas* spp. strain G compared with those in plants inoculated with the pathogen alone. The disease reduction was dependent on endophyte interference with diffusible signal factor (DSF)-mediated signaling. Specifically, the highest suppression level was obtained using *carAB* mutants of *Pseudomonas* spp. strain G characterized by superior capabilities of DSF degradation (Newman et al., 2008). Lindow et al. (2005) confirmed the ability of endophytic species within the genera *Paenibacillus*, *Pseudomonas*, *Staphylococcus*, and *Bacillus* in reducing disease symptoms by altering *X. fastidiosa* DSF-mediated signaling. A promising result was obtained using the *Burkholderia phytofirmans* strain PsJN against *X. fastidiosa* in different grapevine cultivars co-inoculated or even inoculated 30 days after the pathogen (Lindow et al., 2017). *In planta* experiments suggested that the pathogen limitation could be due to not only molecular interference with *X. fastidiosa* quorum-sensing regulation and biofilm formation but also induction of the grapevine immune defense responses (Lindow et al., 2017). Deyett et al. (2017) found that endophytic *P. fluorescens* and *Achromobacter xylosoxidans* showed significant negative correlations with the *X. fastidiosa* titer; in particular, *P. fluorescens* emerged as a promising BCA of PD.

Phytoplasmas are phloem-limited plant-pathogenic bacteria transmitted by phloem-feeding insect vectors (Dickinson et al., 2013). Different “*Candidatus* phytoplasma” species can infect the grapevine to induce a complex of diseases commonly referred to as grapevine yellows (GYs) (Maejima et al., 2014). The contribution of endophytic *Pseudomonas migulae* 8R6 to inducing resistance to grapevine FD phytoplasma was demonstrated in the experimental host *Catharanthus roseus* by Gamalero et al. (2017). Specifically, the increased resistance was related to the bacterial ACC deaminase activity, which regulates the level of the stress hormone ethylene, leading to lower symptom expression (Gamalero et al., 2017). Bulgari et al. (2011, 2014) reported that the grapevine cv. Barbera recovered from FD showed a higher level of bacterial diversity than infected plants. In particular, some bacterial species associated with induced systemic resistance (ISR), such as *Burkholderia* sp., *Bacillus pumilis*, and *Paenibacillus pasadenensis*, were found only in the recovered but not in the infected grapevines (Bulgari et al., 2011). Moreover, the population dynamics of endophytic bacteria, such as *Burkholderia*, *Methylobacterium*, and *Pantoea*, were influenced by the presence of phytoplasma (Bulgari et al., 2014). The presence of ISR-inducing bacteria in the recovered plants could indicate the possible involvement of endophytes in recovery from GYs.

### Protection Against Fungal Pathogens

Recently, several new potential BCAs were identified by culture-dependent techniques coupled with the *in vitro* assessment of fungal inhibition. Some endophytes isolated from Glera grapevine have been shown to produce biocontrol molecules active against phytopathogenic fungi; among them, *Bacillus licheniformis* GL174 was found to secrete cyclic lipopeptides (LPs) belonging to the surfactins and lichenisins families (Favaro et al., 2016; Nigris et al., 2018). Andreolli et al. (2016) isolated 11 bacterial strains within the genera *Bacillus*, *Brevibacillus*, *Lysinibacillus*, *Nocardioides*, *Stenotrophomonas*, *Microbacterium*, *Pantoea*, and *Pseudoxanthomonas* from field-grown grapevines cv. Corvina, showing *in vitro* antifungal activity against the necrotrophic pathogen *B. cinerea*, which is the agent of grapevine gray mold. The ability of endophytic *Bacillus* spp. to induce resistance against *B. cinerea* in grapevine was also reported by Farace et al. (2015), who showed that grapevine plant cells perceive three families of cyclic LPs from *B. subtilis* that differentially activate the plant innate immune response. Campisano et al. (2015) isolated 25 endophytic bacterial strains showing high inhibitory effects against *B. cinerea* from domesticated and wild grapevines in northern Italy. Among them, the most effective strains belonged to the genera *Bacillus* and *Pantoea*.

Several works proved the effectiveness of *Pseudomonas* spp. as BCAs against *B. cinerea*. *P. fluorescens* PTA-CT2 can elicit defense responses in grapevine against *B. cinerea* at both the local and systemic levels (Trotel-Aziz et al., 2008; Verhagen et al., 2011; Gruau et al., 2015). Although PTA-CT2 can colonize grapevine roots but not above-ground plant organs, a systemic defense response is activated by molecular signals transferred from the roots to distal leaves (Gruau et al., 2015). Distinct patterns of defense-related gene expression were found in grapevine roots and leaves, particularly in some genes associated with cell death and hypersensitive response (HR) (Gruau et al., 2015). Following *B. cinerea* infection in grapevine leaves, PTA-CT2-mediated ISR enhanced stilbene accumulation, glutathione 3-transferase gene expression, the downregulation of HR and cell death marker genes (Gruau et al., 2015). Verhagen et al. (2010) found that the resistance to *B. cinerea* in grapevine could be induced by *Pseudomonas aeruginosa* (7NSK2), *P. fluorescens* (strains CHA0, Q2-87 and WCS417), and *Pseudomonas putida* (WCS358) by the production of phytoalexins and stimulation of oxidative bursts.

*Streptomyces anulatus* S37 isolated from wild *V. vinifera* is an endophytic PGP rhizobacterium (PGPR) that confers resistance against different pathogens, including *B. cinerea* (Loqman et al., 2009). Vatsa-Portugal et al. (2017) showed that *S. anulatus* S37 perception by grapevine cells triggers early and late defense responses, such as ion fluxes, oxidative burst, extracellular alkalinization, activation of protein kinases, induction of defense gene expression, and phytoalexin accumulation. Moreover, *S. anulatus* S37-primed grapevine cells became refractory to infection by *B. cinerea*, showing a reduction in pathogen-induced cell death (Vatsa-Portugal et al., 2017).

The *Paraburkholderia phytofirmans* strain PsJN is an endophytic PGPR known for its antifungal activity against gray mold disease (Ait Barka et al., 2000). Following root inoculation, bacterial cells diffuse through grapevine xylem vessels, forming a biofilm at the leaf surface and exerting a direct antagonistic effect on *B. cinerea* (Miotto-Vilanova et al., 2016). Different works revealed that the grapevine responds to *P. phytofirmans* PsJN inoculation by the activation of a local immune response characterized by the accumulation of phenolic compounds, salicylic acid (SA) accumulation, ion fluxes, and defense gene regulation (Compant et al., 2005; Bordiec et al., 2011; Miotto-Vilanova et al., 2016). Moreover, following infection by *B. cinerea*, only the bacterized grapevine plantlets trigger an oxidative burst in leaf tissues, callose deposition in the stomata, the induction of SA and jasmonic acid (JA) pathogenesis-related (PR) genes (*PR1*, *PR2*, and *PR5*) and changes in leaf carbohydrate metabolism (Miotto-Vilanova et al., 2016). In particular, *P. phytofirmans* is associated with the ability to redirect carbohydrates in favor of fructose, which is actually not useful for the fungus (Miotto-Vilanova et al., 2016).

Grapevine trunk diseases (GTDs), such as Esca, Eutypiosis, and Botryosphaeriae diebacks, are associated with a complex of fungal species causing premature decline in vines, yield loss, and poor wine quality worldwide (Gramaje et al., 2018). Pruning and fungicide treatment applied to control GTDs have limited efficacy; moreover, these approaches are expensive and not environmentally sustainable. Eutypa dieback, which is caused by the Diatrypaceous fungus *Eutypa lata*, is a major trunk disease in grapevines associated with the heavy loss of production (Gramaje et al., 2018). Ferreira et al. (1991) reported that an endophytic strain of *B. subtilis* isolated from grapevine cv. Chenin Blanc inhibited *E. lata* mycelium growth and spore germination *in vitro* and significantly reduced fungal infection on pruning wounds. Several endophytic bacterial strains were investigated due to their inhibitory effect on *Phaeomoniella chlamydospora* and *Phaeoacremonium aleophilum*, which are the agents of grapevine Esca disease colonizing the xylem tissues of vine plants (Gramaje et al., 2018). The *B. subtilis* strain AG1 was reported to inhibit mycelial growth of *P. chlamydospora* and *P. aleophilum in vitro* by producing antagonistic substances (antibiotics) stable at high temperatures and resistant to enzymatic degradation (Alfonzo et al., 2009). Another interesting example includes *Paenibacillus* sp. (S19) and *Bacillus pumilus*, which possess effective antagonistic activity against *P. chlamydospora* through the production of the volatile compound pyrazine, which inhibits mycelia growth (Haidar et al., 2016b). Similarly, two *Bacillus* spp. strains effective against Esca-associated fungi were isolated from field-grown grapevines cv. Corvina (Andreolli et al., 2016). More recently, the same research group identified a *Pseudomonas protegens* MP12 strain in a soil sample able to colonize inner grapevine tissues and is exhibiting *in vitro* inhibitory effects on mycelial growth of *P. chlamydospora* and *P. aleophilum* (Andreolli et al., 2019). This strain showed *in vitro* activity against several grapevine phytopathogens, such as *B. cinerea*, *Alternaria alternata*, *Aspergillus niger*, *Penicillium expansum*, and *N. parvum*, and *in vivo* antifungal activity against *B. cinerea* on grapevine leaves (Andreolli et al., 2019). Endophytic bacteria showing antagonistic activity against *N. parvum*, which is among the most virulent GTD-associated fungi, were isolated from grapevine wood tissues in Tunisia (Rezgui et al., 2016; Haidar et al., 2016a). Among 11 strains showing *in vitro* activities against *N. parvum*, *B. subtilis* B6 reduced the size of wood necrosis on young grapevines cv. Italia (Rezgui et al., 2016). Several endophytic fungi have also been proposed for the biocontrol of grapevine truncal diseases; these fungi include *Pythium oligandrum* as an inducer of plant resistance (Yacoub et al., 2016), *Aureobasidium* spp. (Gonzàlez et al., 2012), *Chaetomium* spp. (Spagnolo et al., 2012), *Fusarium lateritium* (Christen et al., 2005), *Trichoderma atroviride* (Pertot et al., 2016), and *Epicoccum layuense* (Del Frari et al., 2019)

Recently, Álvarez-Pérez et al. (2017) demonstrated the efficacy of Actinobacteria isolated from the grapevine root system as BCAs of GTDs. Field trials enabled the identification of the endophytic strain *Streptomyces* sp. VV/E1, which signiﬁcantly reduced the infection rates of *Dactylonectria* sp., *Ilyonectria* sp., *P. chlamydospora*, and *Phaeoacremonium minimum* and is associated with a decline in young grapevines (Álvarez-Pérez et al., 2017).

Wicaksono et al. (2016) reported the potential role of endophytic bacteria from roots of *Leptospermum scoparium*, a New Zealand native medicinal plant, as BCA against botryosphaeriaceous species of fungi associated with GTDs. *In vitro* assays of 10 *Burkholderia* spp., *Serratia* sp. and *Pseudomonas* spp. showed that all isolates were effective against *Neofusicoccum* spp. by the production of antibiotic diffusible and/or volatile compounds (Wicaksono et al., 2017). *Pseudomonas* spp. isolates from *L. scoparium*, which are active against multiple botryosphaeriaceous species *in vitro*, showed evidence of specificity towards a particular pathogen species once inoculated *in planta* (Wicaksono et al., 2017).

Grapevine downy mildew caused by the oomycete *Plasmopara viticola* is a serious and persistent disease problem for the grapevine industry that is difficult to control through chemical and agricultural practices. Musetti et al. (2006) demonstrated the antifungal activity against *P. viticola* of diketopiperazines (DKPs) produced by an endophytic strain of *A. alternata* isolated from grapevine leaves showing anomalous downy mildew symptoms. The application of DKPs inhibited *P. viticola* sporulation and induced severe ultrastructural alterations on fungal mycelium (Musetti et al., 2006). *Acremonium byssoides* is an endophytic fungus naturally present in different grapevine varieties (Burruano et al., 2008). *A. byssoides* is able to actively parasitize the pathogen in grapevine leaves inoculated with *P. viticola*. Moreover, culture filtrates and a crude extract of an *A. byssoides* strain isolated from grapevine cv. Insolia completely inhibited sporangial germination of *P. viticola* (Burruano et al., 2008). In a recent study, *B. subtilis* GLB191 and *B. pumilus* GLB197 were identified among 239 bacterial endophytes isolated from grapevine leaves in China (Zhang et al., 2017). Their potential application as BCAs against downy mildew disease was demonstrated by leaf disk assays and under field conditions. The bacterium *Paenibacillus* sp. strain B2 secretes the peptide paenimyxin, which acts as a biopesticide against different grapevine pathogens. Hao et al. (2017) showed that *Paenibacillus* sp. strain B2 can inhibit the development of *P. viticola* and *B. cinereain vitro* and affects the activity of the ectoparasitic nematode *Xiphinema indexin vitro* and *in planta*.

### Protection Against Insects

Finally, grapevine protection against piercing-sucking insects was achieved by the endophytic colonization of the entomophagous fungus *Beauveria bassiana* in both young potted grapevine plants in a greenhouse and mature plants in the vineyard (Rondot and Reineke, 2018). Following spray inoculation, endophytic *B. bassiana* was detected for at least 21 days inside the leaves of potted plants and up to 5 weeks after the final application in the field. Experimental trials demonstrated that the endophytic fungus can reduce the infestation rate and growth of the mealybug vector of leafroll and rugose wood viruses *Planococcus ficus* in leaves of potted grapevines and infestation of the grape leafhopper *Empoasca vitis* in the field (Rondot and Reineke, 2018).

## Influence of Endophytes on Product Quality

Among the plant products, wine entails one of the most rewarding items in the current agricultural economy. Its market value attracts exceptional attention to the plant’s overall conditions, including its inner microbiome. Since endophyte metabolism can contribute to that of the plant host and the biochemical composition of its fruits, the nature of grapevine endophytic taxa identities, ecological attitudes, potential toxicity, and clinical relevance are all aspects worthy of a thorough investigation.

While the beneficial effect of endophytes on host plants as growth promoters and stress resistance inducers has been reported in several papers, only a few studies addressed their influence on grape and wine quality and almost exclusively focused on fungal endophytes. Recent works demonstrated that wine and its bouquet are also under the influence of endophytic colonizers of grapes. Yang et al. (2016) showed that the inoculation of eight fungal endophytes isolated from *Vitis vinifera* modified the physio-chemical status of field-grown grapevines in both leaves and berries during the ripening stage. In particular, fungal endophytes induced variations in the content of reducing sugar, total flavonoids, total phenols, trans-resveratrol, and activities of PAL in both tissues. Moreover, the inoculation of different strains of fungal endophytes led to different grape metabolite statuses with some strains, such as CXB-11 (*Nigrospora* sp.) and CXC-13 (*Fusarium* sp.), exerting greater promotion effects on grapevine metabolites (Yang et al., 2016). Huang et al. (2018) observed that dual cultures with different endophytic fungal strains were characterized by different metabolite compositions in grapevine flesh cells. In particular, the modification of metabolic profiles by fungal endophytes was fungal strain/genus specific. These works confirmed that endophyte–host metabolic interactions influence the introduction of specific metabolites in the host plant, supporting the possibility of using fungal endophytes to shape grape qualities and characteristics (Huang et al., 2018).

The capability of endophytic fungi to produce plant secondary metabolites or phytochemicals could be exploited for cost-effective large-scale production (Suryanarayanan et al., 2009). Over the last few years, different works reported the identification of endophytic fungal strains producing pharmaceutically valuable compounds with beneficial effects on human health, such as resveratrol. Grapevine inoculation with endophytic *Acinetobacter lwoffii*, *Bacillus subtilis*, and *Pseudomonas fluorescens* effective against *Botrytis cinerea* leads to the accumulation of host-synthesized stilbenic phytoalexins, especially trans-resveratrol (3,5,4′-tryhydroxystilbene) and its oligomer, trans-ε-viniferin, which, in turn, could contribute to the grape fruit metabolite composition (Verhagen et al., 2011). Recently, 36 endophytic fungal strains isolated in China from grapevine cv. Cabernet Sauvignon were assessed for their ability to produce resveratrol *in vitro* (Liu et al., 2016). The morphological and molecular analyses allowed the identification of the C2J6 strain of *Aspergillus niger*, showing stable high resveratrol production (Liu et al., 2016). Dwibedi and Saxena (2018) analyzed the resveratrol-producing potential of 53 endophytic fungi from different *V. vinifera* varieties in different regions in India. The resveratrol-producing isolates were assigned to the following seven genera: *Aspergillus*, *Botryosphaeria*, *Penicillium*, *Fusarium*, *Alternaria*, *Arcopilus*, and *Lasiodiplodia*. In particular, the highest resveratrol content was obtained from the culture of *Arcopilus aureus* isolate #12VVLPM (Dwibedi and Saxena, 2018).

In conclusion, far from being the ultimate framing of such a dynamic matter, this article describes the current picture of this entangling, paradigmatic, and multi-faceted example of a wealthy array of finely evolved plant–microbe interactions.

## Author Contributions

DP, AS, FC, and MZ wrote the first draft. DP, AS, DC, EB, FS, RM, FC and MZ made substantial, direct, and intellectual contributions to this work.

## Conflict of Interest

The authors declare that the research was conducted in the absence of any commercial or financial relationships that could be construed as a potential conflict of interest.
